# The impact of COVID-19 on systemic anticancer treatment delivery in Scotland

**DOI:** 10.1038/s41416-021-01262-8

**Published:** 2021-02-02

**Authors:** Mark A. Baxter, John Murphy, David Cameron, Judith Jordan, Christine Crearie, Christina Lilley, Azmat Sadozye, Mary Maclean, Peter Hall, Angela Phillips, Alex Greger, Jude Madeleine, Russell D. Petty

**Affiliations:** 1 Division of Molecular and Clinical Medicine, Ninewells Hospital and Medical School, University of Dundee, Dundee, UK; 2 Tayside Cancer Centre, Ninewells Hospital and Medical School, NHS Tayside, Dundee, UK; 3 NHS Lanarkshire, South Lanarkshire, UK; 4 Edinburgh Cancer Research Centre, University of Edinburgh, Edinburgh, UK; 5 Edinburgh Cancer Centre, NHS Lothian, Edinburgh, UK; 6 NHS Grampian, Aberdeen, UK; 7 West of Scotland Cancer Network, Glasgow, UK; 8 NHS Highland, Inverness, UK

**Keywords:** Oncology, Chemotherapy

## Abstract

Understanding the impact of the COVID-19 pandemic on systemic anticancer therapy delivery (SACT) is crucial to appreciate the short- and long-term consequences for cancer patients and plan future care. Here, we report real-time national SACT delivery data from NHS Scotland. We demonstrate an initial rapid reduction in patient attendance of 28.7% with subsequent rapid recovery following service redesign. The smallest decrease was seen in breast cancer (19.7%), which also had the most rapid recovery and the largest decrease seen in colorectal cancer (43.4%). Regional variation in the magnitude of impact on SACT delivery was observed, but nadirs occurred at the same time and the rate of recovery was similar across all regions. This recovery reflected a coordinated national approach and associated patient and clinician support structures, which facilitated the creation of COVID-19-protected areas for SACT delivery in Scottish cancer centres enabling rapid sharing of successful and innovative strategies. The data show that these actions have limited the disadvantage to cancer patients.

## Background

COVID-19 (SARS-CoV-2) has impacted significantly on the delivery of cancer care, including systemic anticancer therapy (SACT), in the United Kingdom (UK). During the pandemic major reductions in cancer screening, diagnostic tests or treatment have been observed.^1^

Early studies suggested that cancer patients were at increased risk of SARS-CoV-2 infection and that SACT was associated with severe COVID-19 outcomes.^2^ In addition, the delivery of SACT while maintaining social distancing is challenging. Patient attendance for treatment increases the risk of transmission of infection for both patients and staff. These factors led to uncertainty for cancer clinicians.^3^

In response, the National Institute of Clinical Excellence (NICE) produced a guideline regarding the delivery of SACT for England, while in Scotland, the Scottish Government introduced interim governance arrangements for cancer medicines.^4,5^

UK cancer units adapted and clinicians consulted with patients regarding the potential risk of continuing treatment. Focus turned to immunosuppressive regimes and immune checkpoint inhibitors due to the risk of neutropenia and cytokine storm, respectively.^6^ Ultimately, these adaptations led to a reduction in delivery of SACT. In England and Northern Ireland, data suggested that patient attendances for SACT reduced initially by 45–66%.^7^

The need for evidence to guide decisions led to the development of prospective observational studies such as the UK Coronavirus Cancer Monitoring Project (UKCCMP).^8^ UKCCMP has rapidly collected data with weekly reports provided to 96 participating centres.

The UKCCMP alongside the US COVID-19 and Cancer Consortium (CCC19)^9^ have recently demonstrated that outcomes in cancer patients with COVID-19 are largely driven by age, gender and comorbidity. No detrimental effect of SACT was observed on patient outcomes. These new data provide welcome reassurance that SACT including chemotherapy should be offered to patients if possible. However, these studies had short follow-up time and their retrospective nature led to high proportions of missing data and incomplete adjustment for confounders, and consequently many unanswered questions.^10^

Knowledge of the extent of the impact COVID-19 has on cancer treatment delivery is important for future planning, and to minimise disadvantage to patients in the event of a resurgence of infection. In this rapid short report, we present real-time nationwide data from Scotland on cancer patient attendance for SACT during the COVID-19 pandemic. We demonstrate the impact on a regional and national level and discuss reasons for patterns seen and conclusions for future SACT delivery during the ongoing COVID-19 pandemic.

## Methods

Cancer care in NHS Scotland is delivered across 14 geographic health boards and coordinated by three Regional networks: North of Scotland Cancer Alliance (NCA, population 1,396,780), West of Scotland Cancer Network (WoSCAN, population 3,158,940) and South East Cancer Network (SCAN, population 1,509,500). In all Health boards, SACT is prescribed using the same electronic prescribing system, ChemoCare® (CIS Oncology Ltd, Belfast, UK). This enables data on the number of patients attending for SACT to be extracted rapidly and in real time.

Following the UK-wide lockdown on 23 March 2020, SACT patient attendance and treatment episode (a single SACT administration, reflecting the instances where a patient may attend once but receive more than one treatment such as intravenous (IV) and oral (PO) SACT) numbers were collated weekly from each health board and grouped by the responsible regional Cancer Network, for discussion and review at the national level. Patient attendance for 6 weeks or treatment episodes for 8 weeks prior to lockdown were collected for comparison. The *χ*^2^ test was used to compare differences in patient attendances or treatment episodes. A two-sided *p* value of <0.05 denoted statistical significance. All statistical analyses were done using GraphPad Prism (version 6.0).

## Preprint

A previous version of this manuscript was published as a preprint [27 August 2020].

## Results

**Fig. 1:**
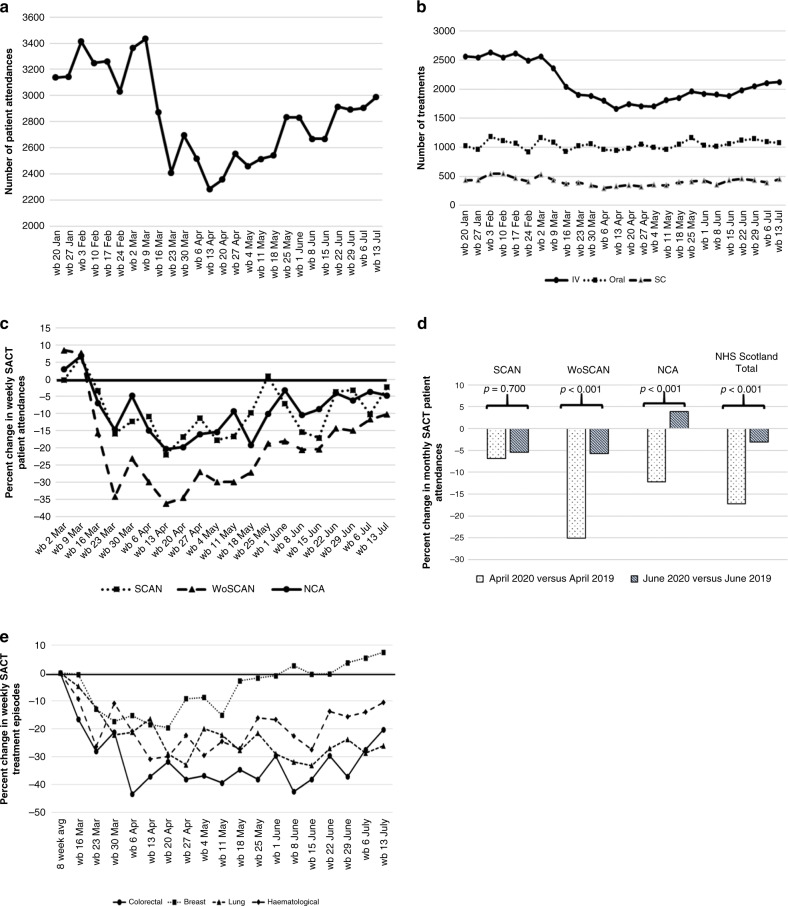
Systemic Anti-Cancer Treatment (SACT) Delivery in Scotland during the COVID-19 Pandemic. **a** Weekly number of patient attendances for systemic anticancer treatment (SACT) in NHS Scotland between the week beginning (wb) 20 January 2020 and the wb 20 July 2020. **b** Weekly SACT treatment episodes in NHS Scotland by route of administration, intravenous (IV), subcutaneous (SC) or oral. **c** Percent change in weekly patient attendances for SACT by regional cancer networks. Percentage change calculated relative to a baseline value determined by the average number of national patient attendances in the 6-week prior to the week beginning 2 March 2020. South of Scotland Cancer Network (SCAN), West of Scotland Cancer Network (WoSCAN) and North of Scotland Cancer Alliance (NCA). **d** Percent change in monthly SACT patient attendance for April 2019 versus April 2020, and June 2019 versus June 2020, by regional cancer networks and in NHS Scotland. **e** Percent change in weekly SACT treatment episodes by tumour type. Percentage change calculated relative to a baseline value determined by the average number of NHS Scotland treatment episodes in the 8 weeks prior to the week beginning 16 March 2020.

Adult patient attendances for SACT in NHS Scotland showed a sudden decrease that was not seen in previous years, in the week preceding the UK lockdown. This decrease continued until the week beginning 13 April 2020 (Fig. 1a). A decrease of 28.7% was seen in the period 2 March to 19 April 2020, by comparison to average weekly attendance in the 6-week period, 20 January to 1 March. Subsequently, a recovery from this point is observed with patient attendances returning towards pre-COVID levels by the week beginning 20 July at −7.5% by comparison to average weekly attendance in the 6-week period, 20 January to 1st March (Fig. 1a). Compared to 2019, attendance in April 2020 decreased by 17.2%, and in June 2020 by 3.1% (*p* ≤ 0.001) (Fig. 1a).

Our national data also enable us to explore regional variation between the three cancer networks (Fig. 1c). Interestingly, a similar fall in patient attendances was seen in SCAN and in NCA, but a greater and more rapid decrease in WoSCAN. The rate of recovery in all regions appears similar with a nadir in the week beginning 13 April (SCAN −21.9%, NCA −20.3% and WOSCAN −36.3%; SCAN versus NCA, *p* = 0.813, SCAN versus WOSCAN, *p* = −0.002, NCA versus WOSCAN, *p* < 0.001), before subsequent recovery.

On assessment of the impact of route, IV, subcutaneous (SC) and PO administration of SACT were all reduced (Fig. 1b). The nadir was observed for all routes of administration in the week beginning 13 April and the decrease was predominantly observed for IV SACT (−34.8%), but a proportionally similar decrease was observed for SC SACT (−30.9%, IV versus SC, *p* = 0.465), and a smaller decrease for PO SACT(−11.24%, PO versus IV, *p* < 0.001, and PO versus SC, *p* = 0.034). (Fig. 1b). This pattern is the same for all regional networks.

The decrease in SACT varied according to tumour type (Fig. 1e). The largest fall in weekly SACT treatment episodes was seen for colorectal cancer (nadir at −43.4% for 6–12 April) and the smallest for breast cancer (nadir at −19.7% for 20–26 April). A rapid recovery in breast cancer SACT was observed (Fig. 1e), with an increase of 17.7% in treatment episodes for the 4-week period 22 June to 19 July, compared to the 4-week period 16 March–12 April (*p* = 0.009). In contrast, for colorectal and haematological cancers, no significant change in SACT treatment episodes was observed (−1.8%, *p* = 0.129 for colorectal cancer, and +4.0%, *p* = 0.452 for haematological cancer), and, for lung cancer, a significant decrease was observed (−13.2%, *p* = 0.038).

## Discussion

In this report, we demonstrate the impact of COVID-19 on SACT delivery in Scotland. The initial decrease observed reflects the uncertainty of the risks associated with SACT at the beginning of the pandemic. This timing pre-dates the UK lockdown indicating an awareness of early, small cohort data from other countries. The subsequent rapid recovery reflects Scottish government interim governance arrangements for cancer medicines that enabled evidence-based, coordinated and transparent quick adaptions to practice based on clinical consensus, and the creation of COVID-19- protected SACT delivery in Scottish cancer centres. Subsequently, larger observational studies, including UKCCMP, provided weekly distribution of real-time data to UK investigators and provided reassurance that SACT should be offered to patients if possible. This generated confidence to sustain the recovery of SACT delivery in NHS Scotland. Overall, these actions, as our data show, have been important in limiting the disadvantage to cancer patients.

A differing impact on SACT delivery was observed at a regional level, with a greater initial decrease seen in WoSCAN. This may reflect the highest incidence of COVID-19 in Scotland being observed in two of the four health boards (NHS Greater Glasgow and Clyde and NHS Lanarkshire) that together comprise 73% of the WoSCAN population. Despite differences in the extent and rate of decrease between regions, the rate of recovery in all regions appears similar. A key reason for this is likely to be the coordinated national recovery approach.

The lesser impact seen on PO SACT may be explained by the drug’s mechanism of action and less need for hospital attendance, which may have encouraged clinicians and patients to continue treatment.

The impact on SACT delivery also varied by tumour site. Greater initial decreases in treatment episodes were observed for colorectal, lung and haematological cancers compared to breast cancer, and the subsequent recovery has been more rapid for breast cancer. The reasons for this may include the different impact on diagnostic modalities or perceived greater risk from COVID-19 in different tumour types. A recent UK-wide analysis, including Scottish centres, demonstrated a reduction in endoscopy, the main modality for diagnosing colorectal cancer to only 5% pre-COVID levels early in the pandemic with a recovery to only 20% 10 weeks later.^11^

The high quality and granularity of NHS Scotland cancer data are enabling further research to allow a deeper understanding of the impact of SACT in different tumour types. This includes when sufficient follow-up has occurred, reporting short- and longer-term outcomes such as cancer-specific survival, and investigating the impact of other factors, for example, any changes in stage at diagnosis due to delayed presentation to healthcare services on outcomes. This analysis will allow ongoing detailed and rapid investigation of the impact of COVID-19 and associated response and recovery of cancer pathway modifications.

A resurgence of SARS-CoV-2 infection has been observed as lockdown restrictions are lifted. The ability to learn from experience will form a key part of future strategies aimed at avoiding any subsequent decrease in patient attendance for SACT if a COVID-19 resurgence occurs. It is evident that not all regions of the United Kingdom have been affected equally in terms of COVID-19 incidence.^12^ Similarly, the impact of SACT delivery may differ according to the administration route. The impact of COVID-19 upon cancer referrals, diagnostic services, and other treatment modalities will also impact SACT delivery, and may disproportionally affect some tumour types. This is the subject of ongoing analysis to ensure the observed recovery in SACT in NHS Scotland is sustained.

## Data Availability

The authors are committed to the responsible sharing of our research data that is consistent with patient consent, protects the confidentiality and meets the highest standards of integrity and ethics. The data are available upon request to the corresponding author indicating the intended objectives and specific details of the use of data and will only be provided consistent with existing ethical approvals and governance standards in place for the data.
