# Correction to: The impact of COVID-19 on systemic anticancer treatment delivery in Scotland

**DOI:** 10.1038/s41416-021-01298-w

**Published:** 2021-03-15

**Authors:** Mark A. Baxter, John Murphy, David Cameron, Judith Jordan, Christine Crearie, Christina Lilley, Azmat Sadozye, Mary Maclean, Peter Hall, Angela Phillips, Alex Greger, Jude Madeleine, Russell D. Petty

**Affiliations:** 1 Division of Molecular and Clinical Medicine, Ninewells Hospital and Medical School, University of Dundee, Dundee, UK; 2 Tayside Cancer Centre, Ninewells Hospital and Medical School, NHS Tayside, Dundee, UK; 3 NHS Lanarkshire, South Lanarkshire, UK; 4 Edinburgh Cancer Research Centre, University of Edinburgh, Edinburgh, UK; 5 Edinburgh Cancer Centre, NHS Lothian, Edinburgh, UK; 6 NHS Grampian, Aberdeen, UK; 7 West of Scotland Cancer Network, Glasgow, UK; 8 NHS Highland, Inverness, UK

**Keywords:** Oncology, Chemotherapy

Correction to: *British Journal of Cancer* 10.1038/s41416-021-01262-8, published online 2 February 2021

The article The impact of COVID-19 on systemic anticancer treatment delivery in Scotland, written by Mark A. Baxter, John Murphy, David Cameron, Judith Jordan, Christine Crearie, Christina Lilley, Azmat Sadozye, Mary Maclean, Peter Hall, Angela Phillips, Alex Greger, Jude Madeleine and Russell D. Petty, was originally published electronically on the publisher’s internet portal on 2 February 2021 without open access. With the author(s)’ decision to opt for Open Choice the copyright of the article changed on 29th January 2021 to © The Author(s) 2021 and the article is forthwith distributed under a Creative Commons Attribution 4.0 International License, which permits use, sharing, adaptation, distribution, and reproduction in any medium or format, as long as you give appropriate credit to the original author(s) and the source, provide a link to the Creative Commons licence, and indicate if changes were made.

The images or other third party material in this article are included in the article’s Creative Commons licence, unless indicated otherwise in a credit line to the material. If material is not included in the article’s Creative Commons licence and your intended use is not permitted by statutory regulation or exceeds the permitted use, you will need to obtain permission directly from the copyright holder.

To view a copy of this licence, visit http://creativecommons.org/licenses/by/4.0.

